# Structural and dielectrical behavior of polypyrrole/huntite composites

**DOI:** 10.55730/1300-0527.3540

**Published:** 2023-01-04

**Authors:** Zeynep TÜRK, Nazlıcan ŞAHİN, Seyfullah MADAKBAŞ, Ferhat ŞEN, Kadir ESMER

**Affiliations:** 1 Department of Computer and Instructional Technologies Education, İstanbul Aydın University, İstanbul, Turkiye; 2 Department of Food Processing, Zonguldak Bülent Ecevit University, Zonguldak, Turkiye; 3 Department of Physics, Marmara University, İstanbul, Turkiye; 4 Department of Chemistry, Marmara University, İstanbul, Turkiye

**Keywords:** Polypyrrole, huntite, composite, dielectrical, thermal properties

## Abstract

In order to characterize stable composites that will contribute to industrial applications, polypyrrole (PPy)/huntite composites were prepared by the physical method. Polypyrrole (PPy)/huntite composites were produced at various huntite concentrations (10, 20, 30, 40 wt.%). Polypyrrole/huntite was characterized by Fourier transform infrared spectroscopy (FTIR), Thermal Gravimetric Analyses (TGA), Differential Thermal Analyses (DTA), Scanning electron microscopy (SEM), and dielectrical properties were investigated by Impedance Analyzer at the room temperature. The FTIR spectra show that huntite mineral coordinate with polymers through carbonate groups. TGA results indicate the main cause of mass loss can be moisture in the samples and unreacted pyrrole monomer. SEM images show that the composites have a granular and spherical geometry. Dielectric measurements showed that at lower frequencies dielectric constants decrease exponentially with an increase in frequency and this behavior indicates that the effect of interfacial polarization becomes more predominant at a lower frequency.

## 1. Introduction

Research has been carried out on conductive polymers for many years. Composite and nanocomposite materials constitute a large part of this research. In these materials, the matrix phase consists of conductive polymers, mainly polypyrrole (PPy), polyaniline, and polythiophene [1–3]. Many composite and nanocomposite materials are currently being developed using PPy. For example, Li et al. [4] prepared polyoxometalate-based metal-organic framework/PPy composites by oxidative polymerization method to be used as electrodes in supercapacitors. It has been reported that the obtained composite electrodes contain abundant active regions with suitable pores and have good conductivity. Xiang et al. [5] remove Cr(VI) ions from the environment by adsorbing, PPy-coated molybdenum disulfide organic-inorganic composites were prepared by in situ oxidative polymerization method. It has been reported that the prepared composites can be used to reduce environmental pollution by adsorbing Cr(VI) ions. In another study, Wang et al. [6] produced PPy/Ag hybrid films under UV irradiation for use as flexible electronic sensors. It has been reported that the obtained sensors perform well in detecting stretching and bending forces, transmitting Morse code, distinguishing changes in acoustic vibrations in normal speech, monitoring respiration and real-time pulse wave under different conditions.

PPy is among the most used polymers in the world in various applications. PPy is frequently used in many electronic applications due to its high conductivity. At the beginning of these electronic applications are conductive composite materials, chemical, and biochemical sensors, electromagnetic shielding, secondary battery, and light-emitting diode [7–10]. PPy can be synthesized electrochemically, through oxidative polymerization, graft copolymerization, or emulsion polymerization [11–13]. The biggest disadvantage of PPy is that it is fragile and does not dissolve. Since PPy does not dissolve easily, it is also very difficult to process [14].

Huntite is a carbonate mineral with the chemical formula Mg_3_Ca(CO_3_)_4_ and it crystallizes in the trigonal system. It is mostly found in nature as a mixture of hydromagnesite and while the chemical formula of huntite is Mg_3_Ca(CO_3_)_4_, the chemical formula of hydromagnesite is Mg_5_(CO_3_)_4_(OH)_2_·4H_2_O. Because of its thermal behavior, huntite mineral is widely used as a flame retardant [15–17]. Many composite studies are carried out by using huntite as an additive. For example, Dike et al. [18] developed composite materials consisting of thermoplastic polyurethane, zinc borate, and huntite. It has been reported that the flame resistance of composites containing zinc borate and huntite is higher than the flame resistance of pure thermoplastic polyurethane, and the best result is obtained by using zinc borate and huntite in equal amounts. In another study, Yurddaskal and Celik [19] produced nanocomposite materials by combining huntite, antimony trioxide, bentonite, and zinc borate nanoparticles in different proportions with polypropylene. It was reported that huntite showed the best flame-retardant performance among huntite, antimony trioxide, bentonite, and zinc borate nanoparticles used at the same rate.

In this study, polypyrrole/huntite composites containing huntite in different ratios were prepared. Structural characterization of the prepared composite materials was carried out with the FT-IR technique. The thermal behaviors of the composites were investigated by TGA/DTA and their morphological structures were examined by SEM. The dielectric properties of the polypyrrole/huntite composites were also determined by an impedance analyzer.

## 2. Experimental

The huntite as a natural raw material was obtained from Denizli/Turkiye as a mixture with the natural mineral hydromagnesite. It is a calcium magnesium carbonate mineral and is included in the dolomite group under the carbonates class. Chemical formula; CaMg_3_(CO_3_)_4_ (15.88% CaO, 34.25% MgO, and 49.87% CO_2_) [20].

Pyrrole, iron (III) chloride, sodium lauryl sulfate, and methanol were purchased from Sigma Aldrich. All chemicals are of high purity and used directly.

### 2.1. Preparation of the composites

Firstly, polypyrrole was synthesized similarly to the method of Rao et al. [21]. Five g (30.8 mmol) of iron (III) chloride was dissolved in 500 mL of distilled water. Sodium lauryl sulfate, 26.65 g (92.4 mmol), was added to the solution and stirred at room temperature for 2 h. The precipitated crystals were filtered and dried at room temperature for 24 h to obtain a yellow-colored iron (III) complex. Then, 5 mL (72.07 mmol) of synthesized polypyrrole and huntite in different formulations (as 10%, 20%, 30%, and 40%, respectively) were mixed in 500 mL methanol for 12 h. At the end of the time, the precipitated polypyrrole (PPy)/huntite composites were filtered, washed several times with distilled water, and dried in an oven at 50°. After that polypyrrole/huntite composite prepared by using huntite at 10% of polypyrrole was coded as PPy10HT. It was coded similarly according to the amount of huntite used in other prepared samples.

### 2.2. Measurements and characterization

FT-IR analyses were performed using Perkin-Elmer Spectrum 100 FT-IR spectrophotometer in the 400–4000 cm^−1^ frequency range.

TGA/DTA analyses were performed using the Mettler Toledo STAR^e^ Thermal Analysis System with a temperature rise of 10° per minute from 30° to 700° under 50 mL argon flow per minute.

SEM analyses were performed using the Phillips XL 30 ESEM-FEG microscope. Prior to analysis, samples were freeze-fractured with liquid nitrogen and their fractured surfaces were covered with a thin layer of gold.

Frequency dependent changes of real and imaginary permittivity and loss factor were investigated using dielectric equations.


ɛ′=CC0,         ɛ″=GwC0,   C0=ɛ0Ad         and         tanδ=ɛ″ɛ′

*C*_0_ is vacuum capacitance, *C* is capacitance, *w* is angular frequency and G is conductance. The dielectric properties of the nanocomposite, measurements were carried out using an impedance analyzer (Wayne Kerr 6500 B Precision; between frequency 40 Hz–100 kHz, UK) at 1 Vrms potential at room temperature.

## 3. Results and discussions

### 3.1. FT-IR spectroscopy of the composites

**Figure 1:**
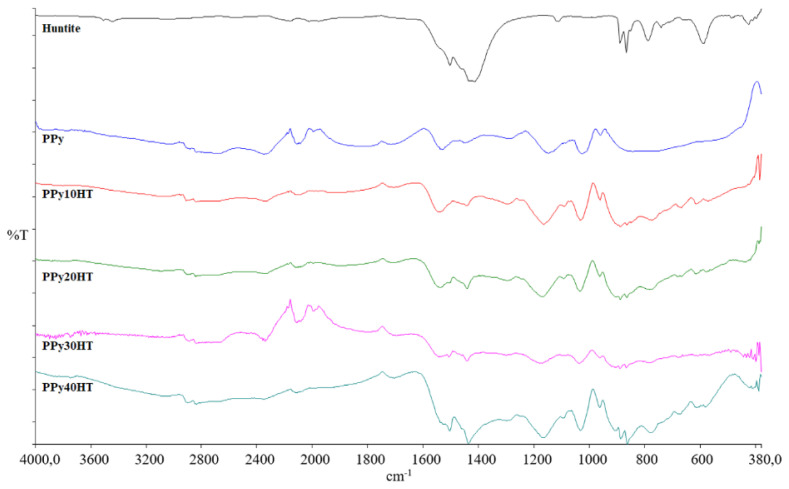
FT-IR spectra of the composites

Figure 1 shows the FT-IR spectra of pure huntite, pure PPy, and composites. When the FT-IR spectrum of pure huntite is examined, bands of magnesium carbonate are observed at 868 cm^−1^ and 891 cm^−1^, and bands of calcium carbonate are observed at 1416 cm^−1^ and 1505 cm^−1^ [15]. When the FT-IR spectrum of pure PPy is examined, peaks belonging to the bands of aromatic C-H bonds are seen at 2840 cm^−1^ and 2910 cm^−1^. In addition, a band of C-C ring tension is observed around 1540 cm^−1^, while a band of C-N ring tension is observed around 1150 cm^−1^. On the other hand, the characteristic band of C-H deformation of PPy is observed at 1025 cm^−1^ [14]. When the FT-IR spectra of the composites are examined, characteristic bands of both PPy and huntite are observed. The FTIR spectra show that huntite mineral coordinate with polymers through carbonate groups. After huntites were added to into PPy the new bands in the spectrum of PPy came about thanks to asymmetric stretching bands of CO_3_^−2^ of huntite [22,23].

### 3.2. Thermal properties of the composites (TGA/DTA)

**Figure 2:**
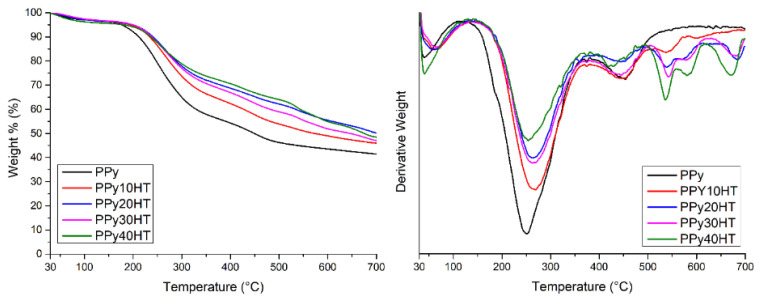
TGA/DTA spectra of the composites.

TGA/DTA technique was used to determine the thermal behavior of the prepared samples. Obtained thermograms from the analysis results are shown in Figure 2. The samples have been dried in a vacuum oven but due to the zeolitic moisture in the huntite structure, It can be said that there is a 5% loss of water at 100 °C in the TGA graph of the samples. The temperatures at which the maximum mass loss is experienced and the remaining % ash amount are listed in Table. When the obtained results are examined, it is seen that there is only a 5% mass loss up to approximately 175° in all samples. This main mass loss can be due to the moisture in the structure of the samples and the unreacted pyrrole monomer. When the main degradation temperatures of the samples are examined, it is seen that the use of huntite causes an increase in the main degradation temperatures of the composites. While the main decomposition temperature of pure PPy was 251 °C, the main decomposition temperature increased to 270 °C with the use of 10% huntite. In addition, the thermal decomposition of the samples in a single step shows that PPy and huntite are in harmony. Especially small losses above 500 °C are due to carbon dioxide released from the decomposition of carbonate ions from huntite [24]. While there is no mass loss in PPy above 500 °C, the DTA graph observing that there is a significant mass loss in the PPy40HT sample containing the highest percentage of huntite supports this situation. When the remaining ash % of the samples at 700° is examined, it is seen that the huntite used causes an increase in the ash amount. While the amount of PPy ash was 41%, this rate increased to 50% in the PPy20HT sample. It can be thinkable that this increase is due to the minerals in the huntite structure.

### 3.3. Morphology of the composites

**Figure 3:**
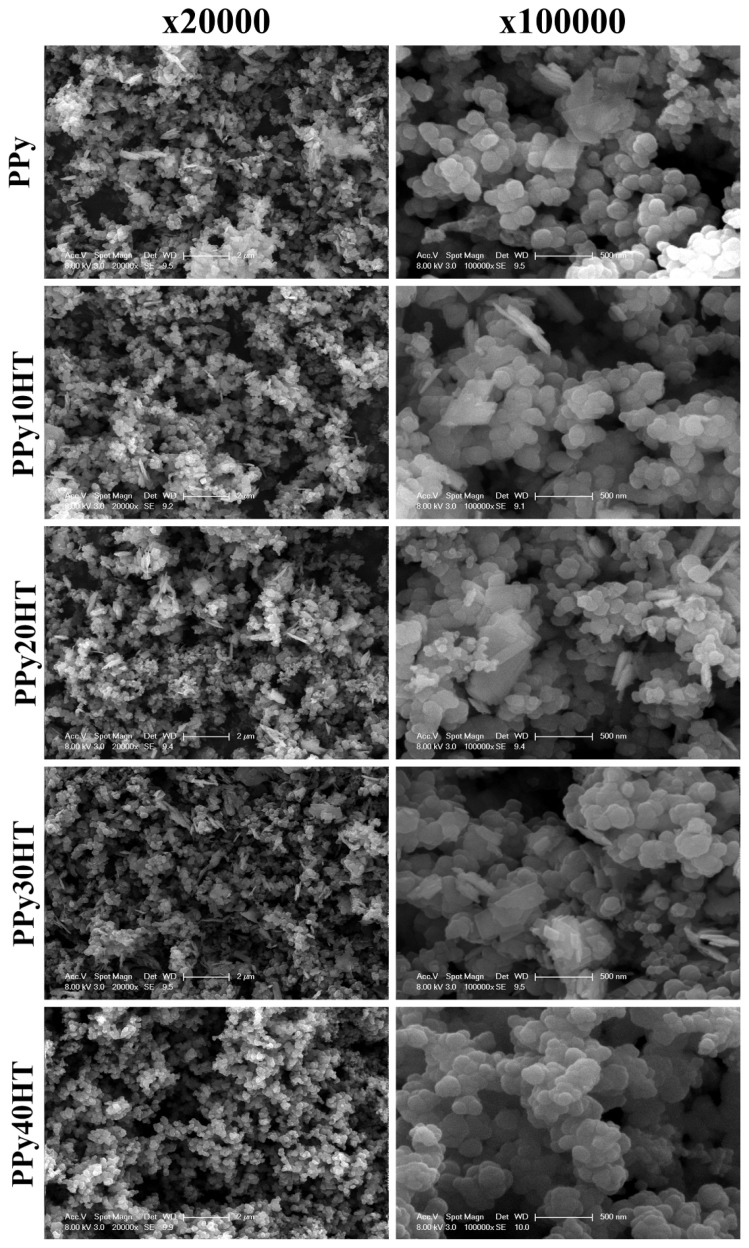
SEM images of the composites

SEM photographs of the composites at 20,000× resolution are shown in Figure 3. For the PPy sample, a 100,000× resolution SEM photograph is also presented. When the SEM photographs are examined, it is seen that the composites have a granular and spherical geometry. In addition, it is seen that as the amount of huntite particules increase agglomerations were observed. Especially this effect is much more visible in the SEM images of 40% huntite containing composites. Dielectric measurements also support the agglomeration behavior and also in good agreement with the SEM finding. It can be said that the 20% huntite contribution is the maximum coordinated to PPy, especially when the mass loss is taken into account according to the amount of huntite.

### 3.4. Dielectrical behavior

**Figure 4:**
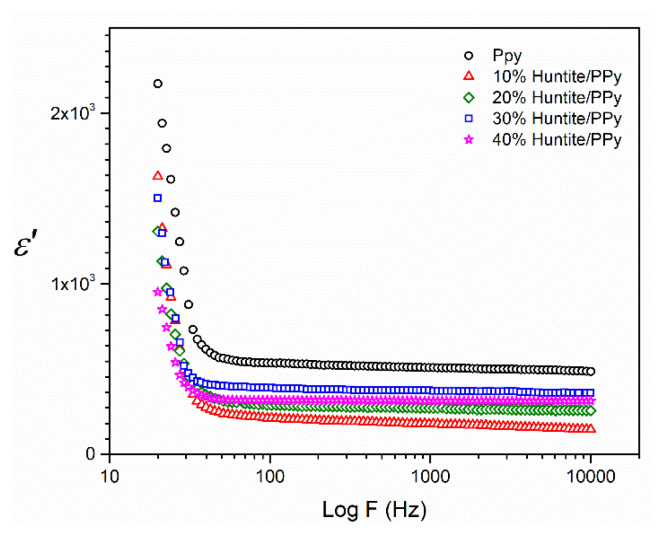
Plots of real values of permittivity versus frequency for PPy/huntite composites.

In Figure 4 the variations of real permittivity with frequency are seen. We can say that the effect of polymer is dominant because of weak molecular mobility between the huntite minerals in the low frequencies’ relaxation and at high-frequency dielectric relaxation should be affected due to orientation of the water molecules in the huntite structure and is more dominant huntite [25]. In the literature, on the studies about polymer–mineral composite/nanocomposites similar changes were observed. It is interpreted that some electrode polarization and interfacial polarization occur at low frequencies, and this situation is dominated by the polymer effect due to weak molecular mobility between mineral layers at low frequency relaxation [26–30].

The factor that most significantly affects the properties of inorganic composites is the interface between the filler and the polymer matrix. Therefore, charge carriers can come up at the interfaces of the composite, causing some space-charge polarization or interfacial polarization. Samet M. et al. have shown in their study that the characteristic frequencies of electrode polarization and of interfacial polarization effects in dielectric spectra of polymer bi-layers are determined and systematically analyzed [31].

**Figure 5:**
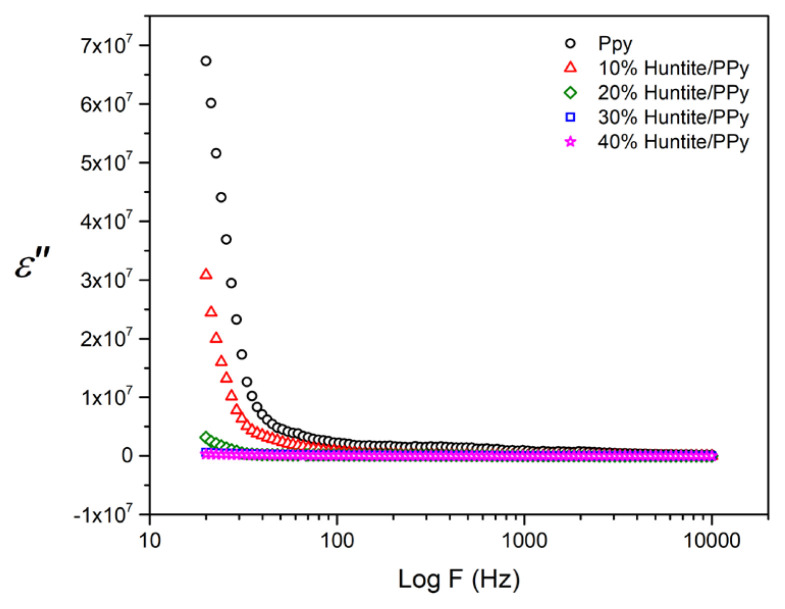
Plots of imaginary values of permittivity versus frequency for PPy/huntite composites.

**Figure 6:**
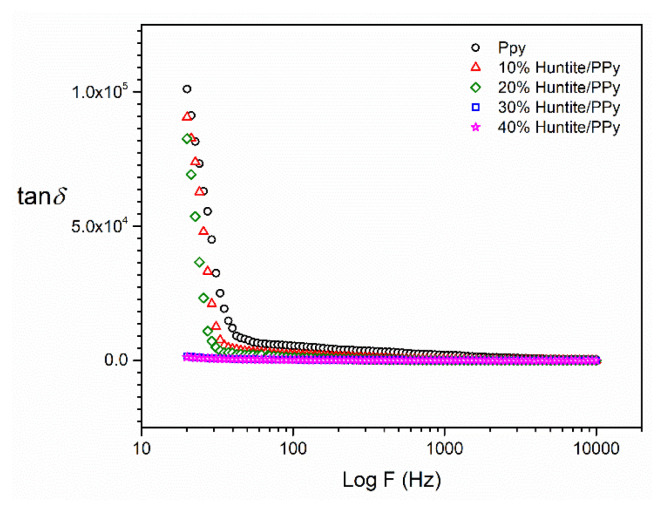
Plots of dielectric loss (tan δ) of permittivity versus frequency for PPy/huntite composites.

In Figures 5 and 6, the variations of imaginer permittivity and tangent loss factor with frequency are shown. Both shapes support each other, and it is seen that the huntite particles are not homogeneously distributed in the polymer structure and there is agglomeration according to the increasing huntite concentration. SEM photographs support this situation and as can be seen from the SEM photographs, it is observed that the huntite particles (at 10% and 20%) are homogeneously dispersed in the matrix, the particles are surrounded by the polymer, and there is no agglomeration.

As seen in Figure 5 the imaginary part of the complex permittivity of PPy decreases gradually with the increasing contribution of the huntite particles. This situation can be interpreted that the reduction of ɛ″ is an expected property from the point of view since low ɛ″ corresponds to low energy loss in the composite. At lower frequencies, dielectric constants decrease exponentially with an increase in frequency. This behavior can be interpreted as the effect of interfacial polarization becoming more and more dominant at lower frequencies [32–33].

As seen in Figure 6, charge mobility is higher in 10% and 20% huntite added composites. Especially at lower frequencies, the effect of orientation polarization of dipoles is higher for getting sufficient time to orient them, thus indicating higher dielectric loss at lower frequencies [33–34].

In the literature, studies on polymer–mineral composites observed similar changes and is expressed that at lower frequencies some electrode polarization and interfacial polarization occur [26–28].

## 4. Conclusion

In this study, PPy/huntite composites were prepared. It has been proven that the composites are prepared successfully with the characteristic bands of PPy and huntite from the FT-IR results of the composites. From the TGA/DTA results of the composites, it was determined that huntite increased thermal strength and % ash content of polypyrrole. The SEM photographs, it is observed that the huntite particles (at 10% and 20%) are homogeneously dispersed in the matrix, the particles are surrounded by the polymer, and there is no agglomeration. It can be said that PPy/huntite composite materials exhibit relaxation mechanisms and dielectric loss at low frequencies. When all the results are evaluated as a whole, it can be said that these new composite materials with high thermal stability can be used in electronic circuits.

## Figures and Tables

**Table:** Thermal properties of the composites.

Sample	T_5%_ (°C)	T_10%_ (°C)	T_max. weight loss_ (°C)	Char yield (%)
PPy	177	212	251	41.40
PPy10HT	195	234	270	45.93
PPy20HT	199	241	263	50.17
PPy30HT	200	239	264	47.06
PPy40HT	182	237	254	48.47
